# Identifications of Common Species and Descriptions of Two New Species of *Siphonaria* (Mollusca: Gastropoda) in China †

**DOI:** 10.3390/biology14010103

**Published:** 2025-01-20

**Authors:** Guochen Zang, Jiahui Wang, Peizhen Ma, Cui Li, Ya Chen, Zeyu Tang, Haiyan Wang

**Affiliations:** 1 Department of Marine Organism Taxonomy & Phylogeny, Institute of Oceanology, Chinese Academy of Sciences, Qingdao 266500, China; coco_sdu@163.com (G.Z.); licui@qdio.ac.cn (C.L.); chenya@qdio.ac.cn (Y.C.);; 2 Zhangqiu Bilingual School, Jinan 250200, China; 3 Laboratory for Marine Fisheries Science and Food Production Processes, Qingdao Marine Science and Technology Center, Qingdao 266237, China; 4 State Key Laboratory of Mariculture Biobreeding and Sustainable Goods, Yellow Sea Fisheries Research Institute, Chinese Academy of Fishery Sciences, Qingdao 266071, China

**Keywords:** *Siphonaria*, taxonomy, phylogeny, China coast, new species

## Abstract

The genus *Siphonaria* is a group of limpet-shaped pulmonates living on intertidal rocks along the Chinese coast. Due to the fluctuating intertidal environment, the morphological characteristics of *Siphonaria* shells are reshaped, resulting in morphologically divergent evolution within species and inaccuracy of taxonomic identification without molecular evidence. Large-scale identifications on *Siphonaria* animals combining morphological and molecular evidence are considerably absent. In this study, we sampled 245 *Siphonaria* specimens along the coast of China (from Guangxi to Shandong) and conducted the identification based on morphological characteristics and DNA barcoding data including *16S* ribosomal RNA, *12S* ribosomal RNA, Cytochrome c oxidase subunit I, and Histone 3. The results indicated five *Siphonaria* species, i.e., *Siphonaria japonica* Donovan, 1824; *Siphonaria sirius* Pilsbry, 1894; *Siphonaria atra* Quoy & Gaimard, 1833; and two new species (*Siphonaria petasus* sp. nov. and *Siphonaria floslamellosa* sp. nov., Zang, Ma & Wang). We conducted a detailed comparison of these species and found many morphological differences, with the most distinctive differences being the radial ribs and siphonal ridge. We created a new identification key based on these features, providing new insights into the phylogeny and taxonomy of the genus *Siphonaria*. In addition, the divergence time indicated that species of the *Siphonaria* genus underwent rapid diversification during the late Pliocene to Pleistocene (3.46–1.53 Mya), with climate likely being an important driver of this diversification.

## Simple Summary

The genus *Siphonaria* Sowerby, 1823 is a group of typical pulmonates living on intertidal rocks. The fluctuation in the intertidal environment gives rise to variations in the shells of the *Siphonaria* species, leading to significant uncertainty in species identification using traditional morphological classification methods. In this study, we analyzed 245 *Siphonaria* specimens collected from the Chinese coast based on both morphological and molecular evidence. Our results revealed that five *Siphonaria* species were identified, i.e., *Siphonaria japonica* Donovan, 1824; *Siphonaria sirius* Pilsbry, 1894; *Siphonaria atra* Quoy & Gaimard, 1833; and two new species (*Siphonaria petasus* sp. nov. and *Siphonaria floslamellosa* sp. nov.). Key features for identifying these species were also provided in this study.

## 1. Introduction

The genus *Siphonaria* G. B. Sowerby I, 1823 is one of the oldest extant marine pulmonates, commonly known as “false limpets” [1]. These animals use a specialized mantle cavity as a pulmonary sac for breathing [2]. To date, 116 *Siphonaria* species have been described worldwide [3], but only 4 species are recorded in China, i.e., *Siphonaria japonica* Donovan, 1824; *S. atra* Quoy & Gaimard, 1833; *S. sirius* Pilsbry, 1894; and *S. laciniosa* Linnaeus, 1758, with the validity of *S. laciniosa* still in doubt [4].

Early monographs described *Siphonaria* primarily based on shell morphology [5]. However, *Siphonaria* inhabits intertidal rocky shores worldwide, except the polar regions [6]. This broad distribution and fluctuating intertidal environment make their morphology variable and large-scale systematic taxonomy studies are quite challenging [7]. Some studies focused on local diversity of *Siphonaria* in specific regions. For example, Jenkins analyzed the morphological features and taxonomic status of various *Siphonaria* species from Australia [8,9,10]. Chambers and McQuaid found that both larval developmental modes existed in two subgenera of *Siphonaria*, contradicting a previous hypothesis that species within each subgenus exhibit a single developmental mode [11,12,13,14,15]. With the development of scanning electron microscope (SEM) technology, anatomical studies of *Siphonaria* soft tissues have gained increasing attention. The microscopical features of the gonads, radula, and other parts have provided important clarification for the taxonomy and individual identification of *Siphonaria* species [4,16].

Previous studies on *Siphonaria* species have shown significant variation in shell morphology at both species and geographic levels [17,18,19,20,21]. The intertidal environment, with its complexity and diverse habitats, provides continuous driving force for the formation and maintenance of cryptic species. Moreover, the morphological characteristics of the radula can be influenced by biological developmental stages and predatory behavior, which may mislead the classification studies on taxonomy of *Siphonaria* species [22]. Therefore, species identification based solely on morphology may not be accurate and molecular evidence is a vital supplement. Teske et al. conducted a phylogenetic study of South African *Siphonaria* species using Cytochrome c oxidase subunit I (*COI*) and ATPSβ sequences, hypothesizing that the four *Siphonaria* species are actually four morphological forms of a single species [23]. Giribet et al. used *COI* and *16S* ribosomal RNA (*16S rRNA*) to study the complex population structure of *Siphonaria pectinata* Linnaeus, 1758 [22]. Colgan et al. analyzed five *Siphonaria* species from southeastern Australia using Histone (*h3)* and ITS-2 sequences and found a high distributional differentiation of *Siphonaria* species in the region [24]. Dayrat et al. conducted the first study on the diversity of *Siphonaria* species in the Indo-West Pacific region, combining shell morphology and molecular data (*12S* ribosomal RNA (*12S rRNA*), *COI*, and *16S rRNA*) and confirming 41 species in the genus [7]. As a result, integrative taxonomy, combining multiple lines of evidence, could play a more important role in the delimitation of *Siphonaria* species.

During 2011–2022, we collected *Siphonaria* specimens along the Chinese coast and conducted the species identification based on both morphological and molecular characteristics. Our aims were (a) to provide detailed morphological descriptions of the species of *Siphonaria* in Chinese waters; (b) to conduct a systematic taxonomic study of Chinese *Siphonaria* species using an integrative taxonomic approach.

## 2. Materials and Methods

### 2.1. Sampling

**Figure 1:**
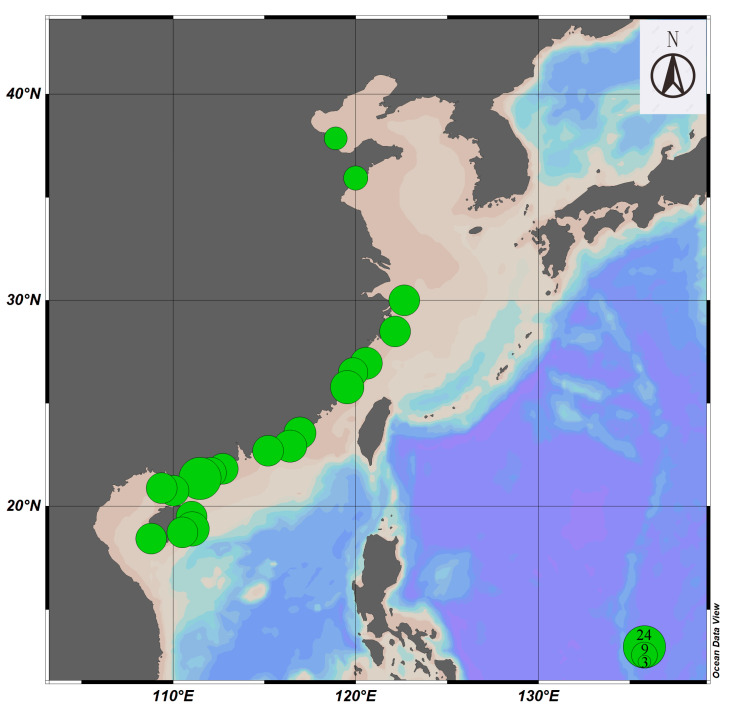
The sampling map of *Siphonaria* species in this study. Note: The size of the circles represents the sample size.

In this study, a total of 245 *Siphonaria* specimens were collected from 19 cities from six provinces (Shandong, Zhejiang, Fujian, Guangdong, Guangxi, and Hainan) along the coast of China (Figure 1, Table A1). All specimens were preserved in 95% ethanol and deposited in the Marine Biological Museum, Chinese Academy of Sciences. The specimens were initially identified based on morphological characteristics such as siphonal ridges, shell size, and radial ribs referring to Bouchet et al. [25] and the World Register of Marine Species database [26].

### 2.2. Microscopic Observation

Shells were observed with a stereomicroscope (Zeiss Stemi SV11, Carl Zeiss AG, Jena, Germany). Morphological indices, including the shell color, size, shape, apex, siphonal notch, etc., were described. Imaging software ZEN 2.3 SP1 was used to capture the images.

### 2.3. DNA Extraction and PCR Amplification

About 30 mg adductor muscle of each specimen was used for DNA extraction using the TIANamp Marine Animal DNA Extraction Kit (DP324, Beijing Tiangen Biochemical Co., Ltd., Beijing, China). We selected three mitochondrial genes (*COI*, *12S rRNA*, and *16S rRNA*) and one nuclear gene (*h3*) for amplification, and all forward primers and reverse primers are shown in Table A2. PCR amplification was performed using the Biometra T100TM thermal cycler (Bio-Rad, Singapore). Reactions were carried out in a 30 μL system containing 15 μL of 2 × Canace^®^ Gold PCR Master Mix (with Dye) (10102ES60, Yeasen Biotech Co., Ltd., Shanghai, China), 12 μL of double-distilled water, 1 μL of each primer, and 1 μL of template DNA. For specimens collected before 2016, Hieff Canace^®^ Gold High Fidelity DNA Polymerase (10149ES10, Yeasen Biotech Co., Ltd., China) was used to perform amplification under the same conditions. The amplification parameters are listed in Table A4. The PCR products were detected by 1% agarose gel electrophoresis, and the resulting bands were sequenced using the 3730XL DNA Analyzer (Tsingke Biotechnology Co., Ltd., Beijing, China).

### 2.4. Phylogenetic Analyses

All sequences obtained in this study were compared with nucleotide sequences in GenBank using the Basic Local Alignment Search Tool (BLAST) to put the specimens into groups (5 groups were decided on in this study) [27]. To further clarify the systematic status of *Siphonaria* specimens in this study, a total of 50 sequences from 16 *Siphonaria* species were downloaded from the National Center for Biotechnology Information (NCBI, Table A3) to construct the phylogenetic trees, with *Bullacta caurina* W. H. Benson, 1842 selected as the outgroup [28]. Given the few *h3* sequences available for *Siphonaria* species in GenBank, the phylogenetic analysis was conducted using *COI*, *12S rRNA*, and *16S rRNA* gene partial sequences. Fourteen specimens, of which the *COI* sequences were successfully obtained, were used for the phylogenetic analysis. The sequences of both those sequenced in this study (42 sequences) and downloaded from GenBank (48 sequences) were edited with BioEdit 7.2.5 and corrected to remove interference from degenerate bases [29]. The sequences were aligned using MAFFT 7 in the normal mode, and conserved regions with unambiguous positional homology were retained using Gblocks 0.91b with default parameters [30]. After alignment, sequences were concatenated, and the best-fit evolutionary model for each partition was selected using ModelFinder based on the corrected Akaike Information Criterion (details in Table A6) [31]. Phylogenetic analyses were conducted using two approaches. Bayesian inference (BI) was performed with MrBayes v.3.2.6, with two independent runs, each comprising four Markov Chain Monte Carlo chains running for 2 million generations and sampling every 1000 generations [32]. The initial 25% of trees were discarded as burn-in after running for 10 million generations. A maximum likelihood (ML) analysis was conducted using IQ-TREE 1.6.8 [33]. All software was integrated into PhyloSuite v. 1.2.2 [34]. Phylogenetic trees were visualized using FigTree v.1.4.3 [35].

### 2.5. Molecular Species Delimitation and Genetic Distance

Considering that only 14 sequences for *COI* were obtained in this study, the genetic distances among 5 *Siphonaria* groups (i.e., *S. atra*, *S. sirius*, *S. japonica*, *S. petasus* sp. nov., and *S. floslamellosa* sp. nov.) were calculated based on concatenations of *12S rRNA*, *16S rRNA*, and *h3* to identify and uncover cryptic species using the Kimura-2-parameter model and analyzed with MEGA X [36,37]. Only specimens with *12S rRNA*, *16S rRNA*, and *h3* successfully sequenced were used here and as a result, 73 concatenations (73 specimens, i.e., *S. atra*, 6; *S. sirius*, 30; *S. japonica*, 16; *S. petasus* sp. nov., 6; *S. floslamellosa* sp. nov., 15) from 219 sequences were used. The phylogenetic relationships of the 73 concatenations were analyzed by constructing a BI and ML tree using the methods described in 2.4. Research has proposed species delimitation methods based on genetic distances [38,39]. Following previous outcomes [40,41,42], we utilized two approaches to determine the molecular species boundaries of *Siphonaria*: ABGD (Automated Barcoding Gap Discovery) and ASAP (Assemble Species by Automatic Partitioning) [43,44]. The concatenated sequences were uploaded to the ABGD web interface [45] and analyzed using the following parameters: a P (prior limit to intraspecific diversity) range of 0.01 to 0.1 and a relative gap width (X) of 1.0. Transition/Transversion Bias (TS/TV, value = 2.0) was estimated using MEGA X, and a data analysis was performed with the Kimura 80 model [36]. The ASAP [46] used the same settings as those used for ABGD.

### 2.6. Haplotype Network

In total, 73 concatenations of *12S rRNA*, *16S rRNA*, and *h3* sequences from 5 species groups were imported into DnaSP v6.12.03 [47]. To generate haplotype networks and visualize the phylogenetic relationships among haplotypes and lineages, PopArt 1.7 was used with the TCS algorithm mapping method [48,49].

### 2.7. Divergence Times

Due to the lack of definitive fossil or geographical record data, this study referred to previous research and selected an average substitution rate of 1.0% per million years to estimate the divergence times of *Siphonaria* species [24,50]. Again, 73 concatenations of *12S rRNA*, *16S rRNA*, and *h3* sequences were used to estimate divergence times. Following Jung et al., we utilized two fossil records to estimate divergence times [51]. These fossil calibrations were based on the estimated ages of the most recent common ancestors as follows: (i) the genus *Siphonaria* (3.6–2.588 million years ago); (ii) *S. pectinata* (0.774–0.129 million years ago). Using the Model Finde in PhyloSuite, the TN93 model was selected for the BEAST analysis [31]. Bayesian MCMC methods in BEAST 2 were employed to estimate divergence times among major lineages using concatenated sequences of *16S rRNA*, *12S rRNA*, and *h3* [52]. The analysis applied an uncorrelated log-normal relaxed clock model combined with a Yule speciation process. Two independent MCMC analyses were conducted, respectively, each running for 50 million generations with sampling every 1000 generations, with the first 25% of samples discarded as burn-in. Convergence of the runs was confirmed using Tracer 1.5 [53], ensuring effective sample sizes (ESSs) exceeding 200. The resulting divergence time tree was then visualized and beautified using Fig Tree v1.4.3.

## 3. Results

### 3.1. Morphological Descriptions of Siphonaria Species in China

This study identified five *Siphonaria* species groups based on morphological characteristics, three of which are known species and two of which are previously unreported. Based on past research and observations of samples, the morphologies of the three known species, i.e., *S. japonica*, *S. atra*, and *S. sirius*, are described as follows [54,55,56,57,58,59,60,61].

#### 3.1.1. *Siphonaria japonica*

**Figure 2:**
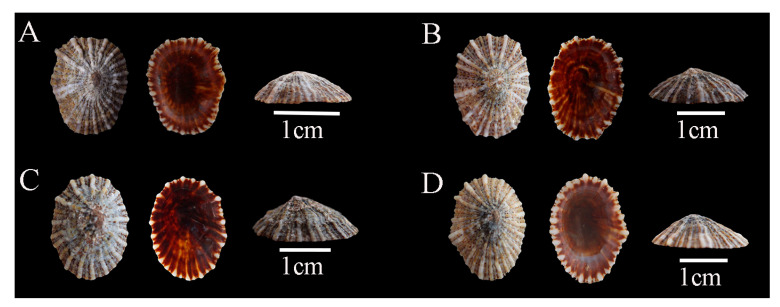
*Siphonaria japonica* specimens collected in Guangdong, China. (**A**), WN-135; (**B**), WN-160; (**C**), WN-446; (**D**), WN-495.

Description: Shell hard and thick, with surface pale yellow or brown; radial ribs more than 20, uniform in thickness; siphonal ridge underdeveloped, directly discernible from neither inside nor outside of the shell (Figure 2).

#### 3.1.2. *Siphonaria sirius*

**Figure 3:**
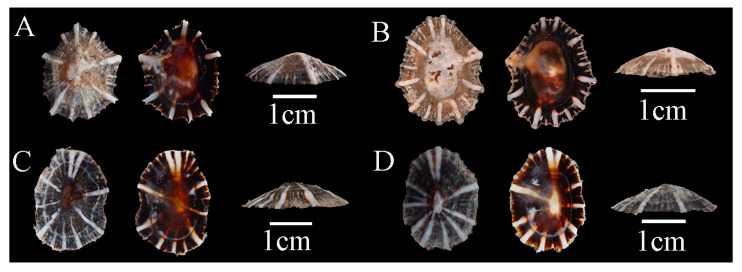
*Siphonaria sirius* specimens collected in Guangdong, China. (**A**), WN-392; (**B**), WN-146; (**C**), WN-148; (**D**), WN-136.

Description: Shell flat, oval- or egg-shaped, relatively thick; shell margin smooth; radial ribs fewer than 20, vary in thickness, slightly raised above the shell surface; apex located at the center; siphonal ridge well developed, allowing direct identification (Figure 3).

#### 3.1.3. *Siphonaria atra*

**Figure 4:**
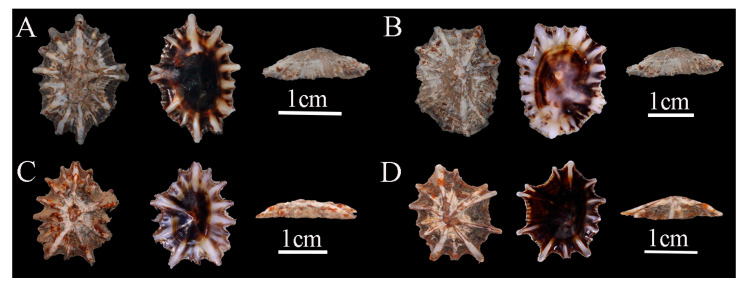
*Siphonaria atra* specimens collected in Hainan, China. (**A**), WN-49; (**B**), WN-47; (**C**), WN-105; (**D**), WN-106.

Description: Shell low, thick, and broad-elliptical; radial ribs fewer than 20, thickness variable, the ends protrude beyond shell margin, giving an irregularly serrated appearance; apex located at the center of the shell; siphonal ridge well developed (Figure 4).

### 3.2. Phylogenetic Relationship

**Figure 5:**
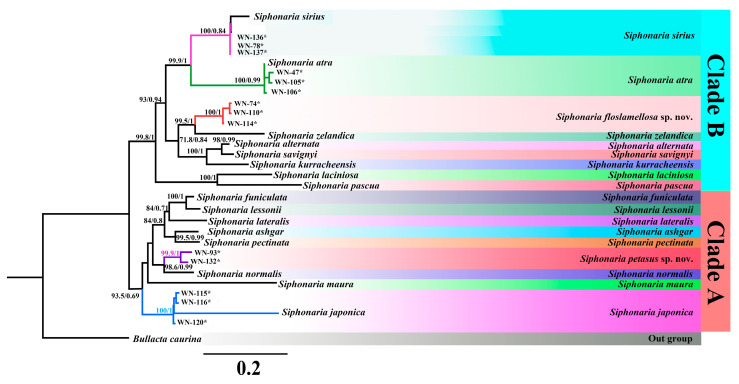
BI tree and ML tree based on concatenated partial sequences of *COI*, *12S rRNA*, and *16S rRNA* of *Siphonaria* species. Maximum likelihood bootstrap support values were followed by Bayesian posterior probabilities, with only posterior probabilities larger than 50% shown. Species marked with asterisk (*) were sequenced in this study.

Totally, 14 of *COI* (648 bp), 127 of *12S rRNA* (317 bp), 142 of *16S rRNA* (423 bp), and 116 of *h3* (303 bp) sequences were obtained in this study (Table A5). Five species groups were decided from all 245 *Siphonaria* specimens by BLAST in this study, corresponding with the morphological results, of which three species were *S. japonica*, *S. sirius*, and *S. atra*. However, sequences of the other two species were quite different from the current sequences in GenBank, suggesting two species have not yet been recognized or studied. The maximum likelihood (ML) and Bayesian inference (BI) phylogenetic trees constructed based on *16S rRNA*, *12S rRNA*, and *COI* revealed similar topologies with most nodes showing high support values (Figure 5). All *Siphonaria* species were divided into two major clades. Clade A included seven known *Siphonaria* species and two species groups obtained in this study, i.e., *S. japonica* (WN-115, WN-116, and WN-120) and *S. petasus* sp. nov. (WN-93 and WN-132). Clade B comprises eight known *Siphonaria* species and three species groups obtained in this study, i.e., *S. atra* (WN-47, WN-105, and WN-106), *S. sirius* (WN-78, WN-136, and WN-137), and *S. floslamellosa* sp. nov. (WN-74, WN-110, and WN-114). Obviously, *S. floslamellosa* sp. nov. formed a distinct clade and clustered with *S. szlandica*. The monophyly of *S. japonica* (posterior probability = 100; bootstrap value = 100), *S. petasus* sp. nov. (posterior probability = 1; bootstrap value = 100), and *S. floslamellosa* sp. nov. (posterior probability = 1; bootstrap value = 100) was strongly supported.

**Figure 6:**
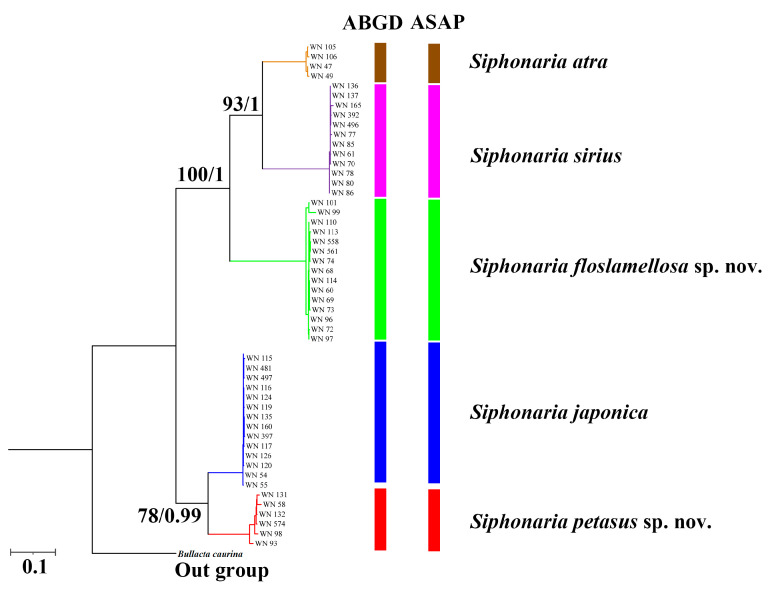
BI tree and ML tree based on concatenated partial sequences of *16S rRNA*, *12S rRNA*, and *h3* gene of *Siphonaria* specimens in this study for species delimitation. Maximum likelihood bootstrap support values were followed by Bayesian posterior probabilities, with only posterior probabilities and bootstrap values greater than 75% shown.

The K2P genetic distances calculated based on concatenated sequences of three fragments are shown in Table 1. The genetic distances between *S. petasus* sp. nov. and the other four species ranged from 11.7% (*S. japonica*) to 21.99% (*S. sirius*), with a mean of 19.17%. The genetic distances between *S. floslamellosa* sp. nov. and the other four species ranged from 16.74% (*S. atra*) to 21.96% (*S. petasus* sp. nov.), with a mean of 19.14%. Both ABGD and ASAP produced consistent species delimitation results, confirming five *Siphonaria* species groups in this study, including the monophyletic units of *S. petasus* sp. nov. and *S. floslamellosa* sp. nov. (Figure 6).

### 3.3. Haplotype Network

**Figure 7:**
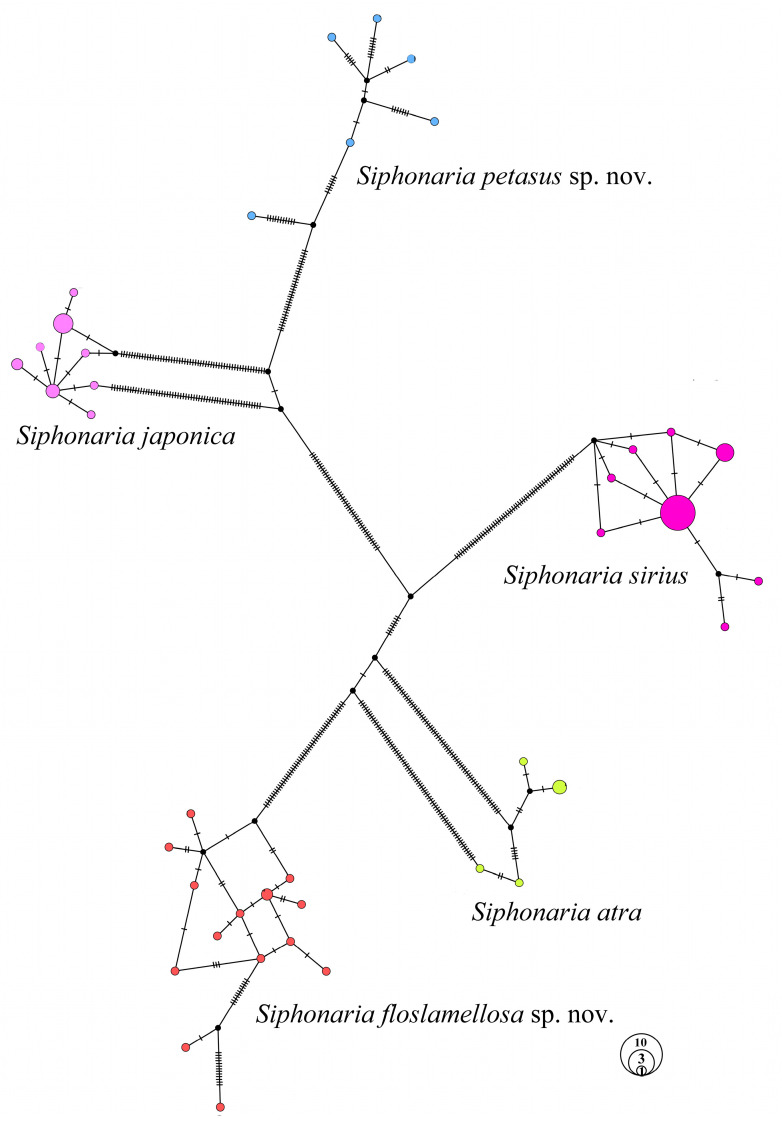
Haplotype network of *Siphonaria* species based on concatenated partial sequences of *16S rRNA*, *12S rRNA*, and *h3*.

The haplotype network constructed by 73 *Siphonaria* samples (Figure 7) revealed that these samples were distinctly divided into five groups, strictly corresponding to the species delimitation. Among them, three known species (*S. atra*, *S. japonica*, and *S. sirius*) exhibited star-like network structures with clearly defined central haplotypes, while the two new species (*S. petasus* sp. nov. and *S. floslamellosa* sp. nov.) displayed complex network structures, with multiple haplotypes interconnected and no distinct central haplotypes identified.

### 3.4. Divergence Time Estimation

**Figure 8:**
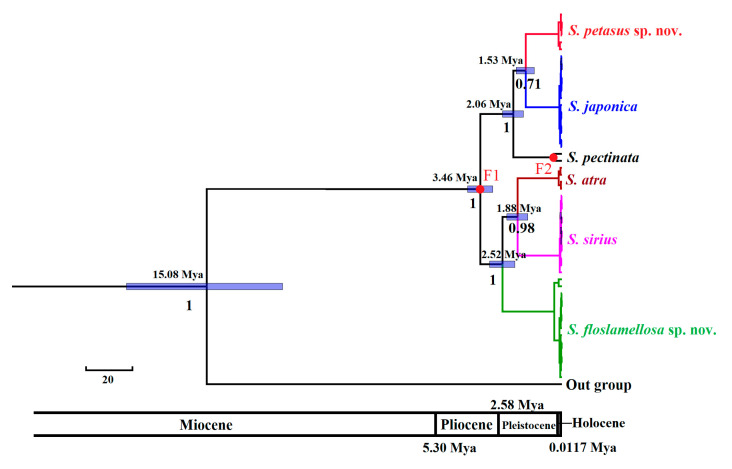
Divergence times of *Siphonaria* species. Node numbers refer to divergence time, with posterior below nodes. Blue shading represents 95% confidence intervals of divergent time. F1: 3.6–2.588 Ma; F2: 0.774–0.129 Ma.

The divergence time estimations (Figure 8) indicated that *S. petasus* sp. nov. and *S. floslamellosa* sp. nov. belonged to different clades, with their divergence occurring at approximately 3.46 Mya (CI_95%_: 2.94–4 Mya) during the late Pliocene. Their common ancestor diverged from *Bullacta caurina*, which also belongs to Tectipleura, approximately 15.08 million years ago (CI_95%_: 11.86–18.48 Mya) during the mid-Miocene. *S. petasus* sp. nov. formed a sister group with *S. japonica*, with an estimated divergence time of about 1.53 million years ago (CI_95%_: 1.17–1.94 Mya) during the early to middle Pleistocene. *S. floslamellosa* sp. nov. formed a sister group with the common ancestor species of *S. atra* and *S. sirius*, with their divergence estimated at approximately 2.52 million years ago (CI_95%_: 2–3.07 Mya) during the early Pleistocene.

### 3.5. Two New Siphonaria Species in China

#### 3.5.1. *Siphonaria petasus* sp. nov.

Taxonomy

Order—Siphonariida J. E. Gray, 1827.

Family—Siphonariidae J. E. Gray, 1827.

Genus—*Siphonaria* G. B. Sowerby, 1823.

Species—*Siphonaria petasus* sp. nov., Zang, Ma & Wang.

Etymology: The name of the species refers to a prominent apex.

Holotype: WN-570. The specimen was collected in May 2012, and deposited in the Marine Biological Museum of the Chinese Academy of Sciences.

Type locality: Wanning, Hainan, China.

Paratypes: WN-102, WN-103 and WN-574. Details are showen in Table 2.

Diagnosis: Shell thin and opaque; apex significantly elevated, slightly inclined to the left, protected by a conical calcareous cover; radial ribs 13 to 18, thickness variable, slightly raised above the surface; shell coloration pale yellow to reddish-brown, apex white.

**Figure 9:**
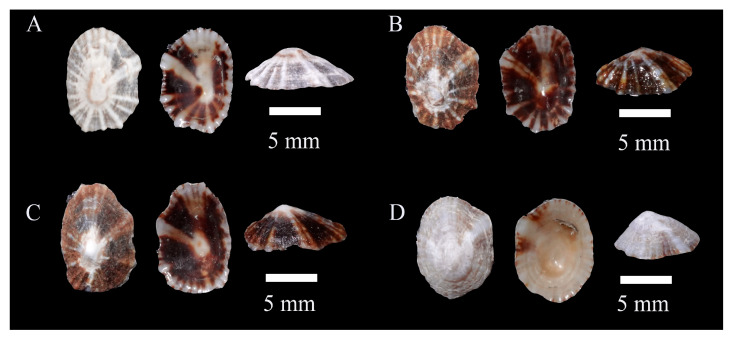
The specimens of *Siphonaria petasus* sp. nov. (**A**), Holotype WN-570. (**B**), WN-102; (**C**), WN-103; (**D**), WN-574.

Description: Shell thin and opaque, diverse coloration, textured, width 5 to 10 mm, length 12 to 16 mm; apex protected by a reinforced conical calcareous cover; radial ribs 13 to 18, thickness variable, extending from apex to shell margin, slightly raised above surface, accompanied by triangular spine-like projections; siphonal ridge distinct, radial ribs pale white; interspaces between ribs vary in color, pale yellow to black, secondary ribs inconspicuous; shell interior deep brown, with a smooth nacreous layer; siphonal notch lighter, pale yellow or white; attachment area of the soft tissues forms continuous muscle scars, interrupted at the siphonal notch, creating a “C”-shaped pattern (Figure 9).

Habitat: Rocky areas of the mid- to low intertidal zones in the eastern and southern regions of Hainan.

Sampling locations: A total of 11 specimens were collected in this study from various locations in Hainan, China, including Wenchang, Sanya, Qionghai, and Wanning (Table A1).

#### 3.5.2. *Siphonaria floslamellosa* sp. nov.

Taxonomy

Order—Siphonariida J. E. Gray, 1827.

Family—Siphonariidae J. E. Gray, 1827.

Genus—*Siphonaria* G. B. Sowerby, 1823.

Species—Siphonaria floslamellosa sp. nov., Zang, Ma & Wang.

Etymology: The species name is derived from the numerous and evenly thick radial ribs, giving the entire shell the appearance of a multi-layered flower.

Holotype: WN-114. The specimen was collected in May 2013, and deposited in the Marine Biological Museum of the Chinese Academy of Sciences.

Type locality: Sanya, Hainan, China.

Paratypes: WN-101, WN-96 and WN-97. Details are showen in Table 3.

Diagnosis: Shell medium to moderately small, irregular, ovate- and cap-shaped, with white calcareous deposits; radial ribs 20 to 30, evenly thick, protrude above the shell surface, extend from apex to margin, ends extend beyond margin; inner shell surface covered with a smooth nacreous layer; right siphonal ridge well developed, muscle scars “C”-shaped, interrupted at the siphonal ridge.

**Figure 10:**
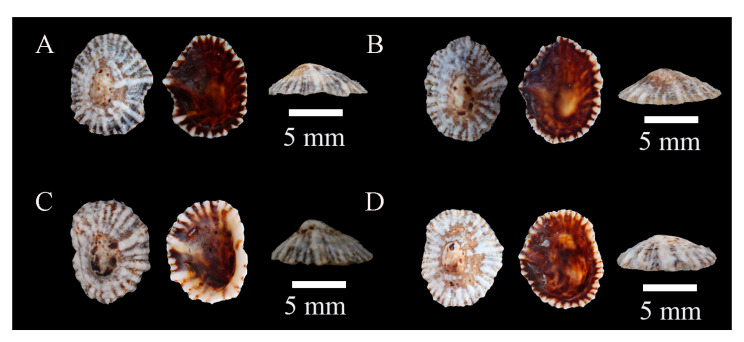
Specimens of *Siphonaria floslamellosa* sp. nov. Holotype: (**A**), WN-114. Paratypes: (**B**), WN-101; (**C**), WN-96; (**D**): WN-97.

Description: Shell medium to moderately small, irregular, ovate- and cap-shaped, width 7 to 11 mm, length 12 to 15 mm; shell thin and opaque, adorned with white calcareous deposits; radial ribs 20 to 30, white, interspaces pale yellow near apex and dark brown toward margin, evenly thick, accompanied by spine-like projections, extending from apex to beyond margin, forming clear ridge-like structures; secondary ribs slender, located between radial ribs; right siphonal ridge well developed; shell apex offset to the left rear, opposite the direction of water canal; inner shell surface covered with a smooth nacreous layer, deep brown; grooves between radial ribs like lighter white bands; muscle scars C-shaped, interrupted at the siphonal ridge (Figure 10).

Habitat: Rocky areas of the mid- to low intertidal zones in the eastern and southern coasts of Hainan.

Sampling location: A total of 25 specimens were collected from various locations in Hainan, including Wenchang, Sanya, Qionghai, and Wanning (Table A1).

### 3.6. Identification Key to Siphonaria Species in China

To facilitate the identification of *Siphonaria* species in Chinese waters, key characteristics of five *Siphonaria* species in China were concluded (Table 4). The key identification for *Siphonaria* species in China was based on characteristics such as the apex, shell margin, radial ribs, and color distribution of the inner shell.

**Identification Key to *Siphonaria* species in China**

1. Radial ribs fewer than 20, varying in thickness, with wide interspaces; radial rib at the siphonal ridge distinctly wider than adjacent ribs······················································2
  Radial ribs more than 20, uniform in thickness, with narrow interspaces; radial rib at the siphonal ridge similar in width to adjacent rib··························································· 3
2. Apex smooth and flat, low, without a covering············································································································································ 4
  Apex elevated, covered with a calcareous layer·····································*S. petasus* sp. nov.
3. The siphonal ridge is underdeveloped; the surface of the shell is pale yellow or brown······················································································································ *S. japonica*
  The siphonal ridge is developed, it can be clearly identified both from the interior and exterior of the shell; the surface of the shell is white············· *S. floslamellosa* sp. nov.
4. Radial ribs flush with shell surface, terminating at the shell edge; shell margin smooth··························································································································*S. sirius*
  Radial ribs significantly raised above shell surface, protruding beyond the shell edge; shell margin irregular and uneven···································································································································*S. atra*

## 4. Discussion

### 4.1. Morphological Analyses

Previous studies suggested that *S. japonica* could be clearly distinguished from *S. sirius* and *S. atra* by its underdeveloped siphonal ridge. Additionally, they proposed that the number of radial ribs could separate *S. sirius* (which typically has six white radial ribs) from *S. atra* (which has more than six radial ribs) [58]. However, this study found that the number of radial ribs is not a stable characteristic within individuals. Some *S. sirius* specimens possess as many as nine or even more radial ribs (Figure 3). Meanwhile, certain *S. atra* individuals have only 6–9 radial ribs (Figure 4). Therefore, relying solely on the number of radial ribs to distinguish them is not reliable. A comprehensive consideration of other morphological characteristics, such as the structure of the radial ribs and the shell margin, should be employed for accurate identification. Meanwhile, we found that the morphological characteristics of radial ribs have been underestimated in previous taxonomic studies. Features of the radial ribs, such as the number, thickness, color at specific locations, whether they extend beyond the shell margin, and whether they rise above the shell surface, can effectively distinguish *Siphonaria* species in China. Radial ribs are among the few traits in *Siphonaria* that can be directly observed with the naked eye without the aid of tools, making them highly efficient for taxonomic research.

In the morphological comparison, we noted that *S. floslamellosa* sp. nov. closely resembles *Siphonaria acmaeoides* Pilsbry, 1894. According to the description by Hirano [60,61], *S. acmaeoides* has a slender, nearly symmetrical shell, thin and opaque, with a calcareous layer covering the shell surface. The radial ribs on the shell surface are light yellow or dark brown, and the interior of the shell is dark brown, with lighter or white areas corresponding to the positions of the radial ribs. The rough radial ribs and the white calcareous covering make these two species appear to be very similar. However, based on the original description by Pilsbry (1894), *S. acmaeoides* has 9–16 radial ribs, and the siphonal ridge is undeveloped, whereas *S. floslamellosa* sp. nov. has 20–30 radial ribs, with a well-developed siphonal ridge. Additionally, *S. acmaeoides* was collected from Japan, while all samples of *S. floslamellosa* sp. nov. were collected from the coastal regions of Hainan Island, China (Wenchang, Qionghai, and Sanya). Therefore, based on morphological differences and distribution records, it can be concluded that *S. floslamellosa* sp. nov. and *S. acmaeoides* are two completely distinct species, and the taxonomic status of *S. floslamellosa* sp. nov. can be confirmed.

### 4.2. Phylogenetic Analyses and Species Delimitation

In this study, we constructed phylogenetic trees using maximum likelihood (ML) and Bayesian inference (BI) methods, based on a short-sequence concatenated dataset of the *COI*, *12S rRNA*, and *16S rRNA* genes (Figure 5). Both the ML and BI trees exhibited consistent topological structures, with clear definitions of the relationships among *Siphonaria* species and extremely high support values. The two species delimitation methods also yielded the same results, both supporting the distinct grouping of *S. petasus* sp. nov. and *S. floslamellosa* sp. nov. (Figure 6). Furthermore, the phylogenetic tree we generated showed branching patterns that are consistent with those of Dayrat, where *Siphonaria* is divided into two major clades, A and B, with *S. petasus* sp. nov. and *S. floslamellosa* sp. nov. assigned to clades A and B, respectively, both of which were strongly supported as monophyletic. A genetic analysis (Table 1) showed that the average K2P genetic distances of *S. petasus* sp. nov. and *S. floslamellosa* sp. nov. from other known *Siphonaria* species with records in China were 18.24% and 18.19%, respectively, which are close to the average interspecies genetic distances of 18.56%, further supporting the validity of these new species. Although the K2P genetic distances between *S. petasus* sp. nov. and *S. japonica* are the smallest (11.7%), there are obvious morphological differences between the two species (Table 4), ruling out the possibility of them being the same species. Unfortunately, due to the lack of fresh samples, we are unable to perform a further mitochondrial genome-based analysis of these species at this time.

It is worth noting that *S. floslamellosa* sp. nov. appears to have been previously studied by Wang [62]. Through sequence alignment, we found that *S. floslamellosa* sp. nov. is identical to the sequences (*COI*, *16S rRNA*) uploaded by Wang to GenBank. However, the article did not explicitly name or describe the species based on morphological features, only noting that it differed molecularly from all known *Siphonaria* species. Therefore, we are unable to directly compare their morphological characteristics and can only infer that they represent the same species based on their highly similar molecular traits.

### 4.3. The Significance of DNA Barcoding in the Taxonomic Study of Marine Mollusca

A large number of reports have utilized DNA barcoding for the species identification of intertidal gastropods. For instance, Sun used *COI* to identify 45 species of Mesogastropoda along the coast of China, and all species were clearly distinguished [63]. Yu et al. used *COI*, *28S* ribosomal RNA, and *h3* to identify *Nipponacmea* species in China, revealing three species: *N. radula*, *N. fuscoviridis*, and *N. nigrans* [64]. However, earlier reports indicated that only *N. schrenckii* existed along the Chinese coast. Similarly, in this study, we found two different shell morphs of *S. petasus* sp. nov.; yet, their DNA sequences were completely identical (Figure 9). These studies demonstrate the significant role of DNA barcoding in the taxonomy of mollusks, as it not only helps researchers quickly and accurately distinguish closely related species, but also provides a thorough understanding of species diversity. This makes DNA barcoding an excellent tool for exploring the hidden diversity within intertidal ecosystems, which are known for their rich cryptic species.

### 4.4. Population History and Dynamics

The divergence time results (Figure 8) indicate that the divergence of the two major clades of the genus *Siphonaria* occurred during the late Pliocene. According to the studies of Lisiecki et al. and Zachos et al., there was a significant global temperature drop and glacial expansion during the Pliocene, laying the foundation for the transition into the Pleistocene ice ages [65,66]. Divergence within each of the two major clades of *Siphonaria* occurred during the Pleistocene. The fluctuations between glacial and interglacial periods during the Pleistocene were particularly intense, with the fluctuation cycles gradually extending from 40,000 years in the early Pleistocene to 100,000 years in the late Pleistocene [67]. During these alternating cycles, sea level changes caused the frequent fragmentation and restoration of intertidal habitats. The Qiongzhou Strait, affected by land bridge formation, periodically opened and closed, which may have directly influenced the isolation and gene flow of intertidal species, such as *Siphonaria*, and subsequently affected the evolutionary processes of *Siphonaria* species.

The haplotype network we obtained (Figure 7) shows that the three species, *S. japonica*, *S. atra*, and *S. sirius*, exhibit a center–periphery structure, with a central, highly common haplotype and several other haplotypes that differ by one to five steps. In contrast, the networks of *S. petasus* sp. nov. and *S. floslamellosa* sp. nov. display both star-like and complex structures. In the haplotype networks of these two species, there are many dispersed haplotypes with very small differences, mostly ranging from one to eight steps. This may be due to the fragmentation of intertidal habitats during the alternation between glacial and interglacial periods, which led to the *Siphonaria* populations undergoing multiple migrations and expansions. Different migration and expansion events superimposed ultimately resulted in the coexistence of star-like and complex branching network structures.

The distribution patterns of *Siphonaria* observed by Dayrat et al. in the Indo-Pacific region are similar to those observed in other gastropod groups, where the ranges of closely related species do not overlap. According to the study by Williams and Reid [68], this suggests that *Siphonaria* in the Indo-Pacific region follows an allopatric speciation pattern. However, in this study, we found that the distribution ranges of the four *Siphonaria* species in the Hainan Island region overlap. This may be due to the rise in sea level during interglacial periods, which led to the restoration of intertidal habitats and the disappearance of geographical barriers. Additionally, ocean currents may have caused the larvae of *Siphonaria* to exhibit migratory behavior in the same direction, ultimately leading to sympatric distributions of different *Siphonaria* species.

## 5. Conclusions

In this study, we described two new *Siphonaria* species collected in China, i.e., *Siphonaria* petasus sp. nov. and *Siphonaria* floslamellosa sp. nov. Through detailed morphological comparisons and a molecular phylogenetic analysis, we confirmed that these species exhibit clear evolutionary divergence from other congeners. We found that the characteristics of radial ribs were highly effective in distinguishing *Siphonaria* species. The number, thickness, and structural relationships of radial ribs with the shell surface and shell margin are of great significance in the taxonomy of *Siphonaria* species. By comparing the commonly found *Siphonaria* species in China, we have compiled a dichotomous key for the efficient identification of different *Siphonaria* species. The discovery of new species not only enhances the species diversity of the genus *Siphonaria*, but also provides new evidence for further studies on species differentiation. Due to the significant influence of environmental factors on the shells of marine mollusks, individuals of the same species can exhibit considerable morphological differences, whereas DNA barcoding remains consistently stable. Therefore, DNA barcode-based studies can improve the accuracy of species identification. Furthermore, as a newly developed method for species delimitation based on genetic distances, the applicability of ASAP was validated in this study. In the prospective research on taxonomy of mollusks, molecular methods should be integrated with morphological results.

## Figures and Tables

**Table 1:** The K2P genetic distances among *Siphonaria* species based on concatenated sequences of *12S rRNA*, *16S rRNA*, and *h3*.

Species	*Siphonaria petasus* sp. nov.	*Siphonaria floslamellosa* sp. nov.	*Siphonaria atra*	*Siphonaria sirius*
*Siphonaria petasus* sp. nov.				
*Siphonaria floslamellosa* sp. nov.	0.2196			
*Siphonaria atra*	0.2103	0.1674		
*Siphonaria sirius*	0.2199	0.1803	0.1569	
*Siphonaria japonica*	0.1170	0.1981	0.1980	0.2020

**Table 2:** Paratypes of *Siphonaria petasus* sp. nov.

Label	Capture Date	Type Locality	Location
WN-102	May 2013	Wenchang, Hainan, China	18.2794° N 110.4290° E
WN-103	May 2013	Wenchang, Hainan, China	18.2794° N 110.4290° E
WN-574	May 2012	Wanning, Hainan, China	18.3503° N 109.2461° E

**Table 3:** Paratypes of *Siphonaria floslamellosa* sp. nov.

Label	Capture Date	Type Locality	Location
WN-101	May 2013	Wenchang, Hainan, China	20.0115° N 110.4329° E
WN-96	May 2013	Sanya, Hainan, China	18.2108° N 109.6544° E
WN-97	May 2013	Sanya, Hainan, China	109.6544° E 18.2108° N

**Table 4:** Key characteristics for five *Siphonaria* species in China.

Characteristic	*S. japonica*	*S. petasus* sp. nov.	*S. floslamellosa* sp. nov.	*S. atra*	*S. sirius*
Radial rib number	More than 20	Fewer than 20	More than 20	Fewer than 20	Fewer than 20
Radial ribs with shell surface	Flush above shell surface	Flush above shell surface	Raised above shell surface	Raised above shell surface	Flush with shell surface
Radial ribs with shell margin	Terminating at the shell edge	Terminating at the shell edge	Terminating at the shell edge	Beyond the shell edge	Terminating at the shell edge
Apex	Smooth, flat, without a covering	Elevated, covered with a layer	Smooth, flat, without a covering	Smooth, flat, without a covering	Smooth, flat, without a covering
Siphonal ridge	Underdeveloped	Developed	Developed	Developed	Developed

## Data Availability

Data are contained within the article.

## Appendix A

**Table A1:** Sampling collection information.

Species	Province	Locality	Quantity	Time
*Siphonaria japonica*	Shandong	Dongying	1	March 2022
		Qingdao	1	July 2022
	Zhejiang	Wenzhou	5	March 2013
		Ningbo	2	May 2017
		Zhoushan	2	June 2017
	Fujian	Zhangzhou	2	August 2017
		Fuzhou	5	May 2017
		Quanzhou	1	May 2017
		Ningde	1	March 2013
	Guangdong	Huizhou	2	April 2018
		Shantou	1	April 2018
		Shantou	1	March 2019
		Yangjiang	5	March 2013
		Yangjiang	11	August 2017
		Yangjiang	4	April 2018
		Jiangmen	27	May 2017
		Jiangmen	3	August 2017
		Jiangmen	19	April 2018
*Siphonaria sirius*	Zhejiang	Wenzhou	14	May 2017
		Zhoushan	1	May 2017
	Guangdong	Shantou	1	April 2018
		Jiangmen	30	April 2018
		Jiangmen	14	August 2017
		Yangjiang	28	August 2017
	Hainan	Wenchang	12	May 2013
		Wanning	1	May 2013
		Sanya	3	May 2013
*Siphonaria atra*	Hainan	Sanya	8	January 2014
		Qionghai	1	May 2013
		Lingshui	1	January 2011
	Guangxi	Beihai	1	April 2018
*Siphonaria petasus* sp. nov.	Hainan	Wenchang	2	April 2018
		Wenchang	4	May 2013
		Sanya	3	May 2013
		Qionghai	1	May 2013
		Wanning	2	March 2012
*Siphonaria floelamellosa* sp. nov.	Hainan	Wenchang	5	May 2013
		Qionghai	2	May 2013
		Wanning	1	May 2013
		Sanya	17	May 2013

**Table A2:** Primers used for PCR amplification in this study.

Marker	Primer Name	Primer Sequences	References
*COI*	LCO1490	5′-GGTCAACAAATCATAAAGATATTGG-3′	[69]
	HCO2198	5′-TAAACTTCAGGGTGACCAAAAAATCA-3′	
*16S rRNA*	16SF	5′-GCCTGTTTATCAAAAACAT-3′	[70]
	16SR	5′-CCGGTCTGAACTCAGATCACG-3′	
*h3*	H3F	5′-ATGGCTCGTACCAAGCAGACVGC-3′	[71]
	H3R	5′-ATATCCTTRGGCATRATRGTGAC-3′	
*12S rRNA*	12SF	5′-AAACTAGGATTAGATACCCTATTAT-3′	[7]
	12SR	5′-GAGGGTGACGGGCGGTGTGT-3′	

**Table A3:** GenBank accession numbers of downloaded sequences.

Species	GenBank Accession Nos.			
	*12S rRNA*	*COI*	*16S rRNA*	*h3*
*Siphonaria japonica* Donovan, 1824	KF001063	KF000759	KF000911	LC384345
*Siphonaria asghar* Biggs, 1958	KF001053	KF000749	KF000901	-
*Siphonaria pectinata* Linnaeus, 1758	KF001067	KF000763	KF000915	-
*Siphonaria lateralis* A. Gould, 1846	KF001076	KF000772	KF000924	-
*Siphonaria funiculata* Reeve, 1856	KF001079	KF000775	KF000927	-
*Siphonaria lessonii* Blainville, 1827	KF001035	KF000731	KF000883	-
*Siphonaria normalis* A. Gould, 1846	KF001090	KF000786	KF000938	-
*Siphonaria maura* G. B. Sowerby I, 1835	KF001107	KF000803	KF000956	-
*Siphonaria pascua* Rehder, 1980	KF001109	KF000805	KF000957	-
*Siphonaria laciniosa* Linnaeus, 1758	KF001020	KF000716	KF000868	-
*Siphonaria zelandica* Quoy & Gaimard, 1833	KF001029	KF000725	KF000877	-
*Siphonaria kurracheensis* Reeve, 1856	KF001052	KF000748	KF000900	-
*Siphonaria savignyi* Krauss, 1848	KF001051	KF000747	KF000899	-
*Siphonaria alternata* Say, 1826	KF001108	KF000804	KF000955	-
*Siphonaria atra Quoy & Gaimard*, 1833	KF001023	KF000719	KF000871	-
*Siphonaria sirius* Pilsbry, 1894	KF001136	KF000832	KF000984	LC384094
*Bullacta caurina* W. H. Benson, 1842	HQ833922	-	HQ833986	HQ834193

**Table A4:** PCR amplification programs in this study.

PCR Master Mix	Marker	Program
2 × Hieff Canace^®^ Gold PCR Master Mix (with Dye)	*COI*	94 °C 5 min, 32 × (94 °C 30 s 46 °C 30 s, 72 °C 50 s), 72 °C 8 min, 4 °C hold
	*16S rRNA*	94 °C 5 min, 32 × (94 °C 30 s, 49 °C 30 s, 72 °C 50 s), 72 °C 8 min, 4 °C hold
	*h3*	94 °C 5 min, 32 × (94 °C 30 s, 52 °C 30 s, 72 °C 50 s), 72 °C 8 min, 4 °C hold
	*12S rRNA*	94 °C 5 min, 32 × (94 °C 30 s, 46 °C 30 s, 72 °C 50 s), 72 °C 8 min, 4 °C hold
Hieff Canace^®^ Gold High Fidelity DNA Polymerase	*COI*	98 °C 3 min, 30 × (98 °C 10 s, 46 °C 20 s, 72 °C 23 s), 72 °C 5 min, 4 °C hold
	*16S rRNA*	98 °C 3 min, 30 × (98 °C 10 s, 46 °C 20 s, 72 °C 15 s), 72 °C 5 min, 4 °C hold
	*h3*	98 °C 3 min, 30 × (98 °C 10 s, 50 °C 20 s, 72 °C 15 s), 72 °C 5 min, 4 °C hold
	*12S rRNA*	98 °C 3 min, 30 × (98 °C 10 s, 46 °C 20 s, 72 °C 15 s), 72 °C 5 min, 4 °C hold

**Table A5:** The GenBank accession numbers for the sequences obtained in this study.

	Specimen	*h3*	*12S rRNA*	*16S rRNA*
*Siphonaria petasus* sp. nov.	WN-58	PQ846807	PQ834496	PQ824544
	WN-93	PQ846806	PQ834497	PQ824545
	WN-98	PQ846805	PQ834498	PQ824546
	WN-131	PQ846804	PQ834499	PQ824547
	WN-132	PQ846803	PQ834500	PQ824548
	WN-574	PQ846802	PQ834501	PQ824549
*Siphonaria floslamellosa* sp. nov.	WN-561	PQ846808	PQ834502	PQ824551
	WN-558	PQ846809	PQ834503	PQ824552
	WN-114	PQ846810	PQ834507	PQ824553
	WN-113	PQ846811	PQ834508	PQ824554
	WN-110	PQ846812	PQ834509	PQ824555
	WN-101	PQ846813	PQ834510	PQ824556
	WN-99	PQ846814	PQ834504	PQ824557
	WN-97	PQ846815	PQ834505	PQ824558
	WN-96	PQ846816	PQ834506	PQ824559
	WN-74	PQ846817	PQ834511	PQ824560
	WN-73	PQ846818	PQ834512	PQ824561
	WN-72	PQ846819	PQ834513	PQ824562
	WN-69	PQ846820	PQ834514	PQ824563
	WN-68	PQ846821	PQ834515	PQ824564
	WN-60	PQ846822	PQ834516	PQ824565
*Siphonaria atra*	WN-542	PQ858636	PQ870139	PQ862295
	WN-539	PQ858637	PQ870140	PQ862294
	WN-106	PQ858635	PQ870138	PQ862293
	WN-105	PQ858638	PQ870141	PQ862296
	WN-49	PQ858639	PQ870142	PQ862297
	WN-47	PQ858640	PQ870143	PQ862298
*Siphonaria japonica*	WN-498	PQ858643	PQ870159	PQ862302
	WN-497	PQ858641	PQ870157	PQ862299
	WN-481	PQ858644	PQ870156	PQ862300
	WN-397	PQ858645	PQ870153	PQ862301
	WN-160	PQ858646	PQ870154	PQ862303
	WN-157	PQ858642	PQ870158	PQ862304
	WN-135	PQ858647	PQ870155	PQ862305
	WN-126	PQ858648	PQ870152	PQ862306
	WN-124	PQ858649	PQ870151	PQ862307
	WN-120	PQ858650	PQ870150	PQ862308
	WN-119	PQ858651	PQ870149	PQ862309
	WN-117	PQ858652	PQ870148	PQ862310
	WN-116	PQ858653	PQ870147	PQ862311
	WN-115	PQ858654	PQ870146	PQ862312
	WN-55	PQ858655	PQ870145	PQ862313
	WN-54	PQ858656	PQ870144	PQ862314
*Siphonaria sirius*	WN-568	PQ858666	PQ870160	PQ862315
	WN-562	PQ858667	PQ870161	PQ862316
	WN-559	PQ858669	PQ870162	PQ862317
	WN-556	PQ858670	PQ870163	PQ862318
	WN-496	PQ858672	PQ870164	PQ862319
	WN-392	PQ858673	PQ870165	PQ862320
	WN-165	PQ858678	PQ870166	PQ862321
	WN-164	PQ858679	PQ870167	PQ862322
	WN-185	PQ858674	PQ870168	PQ862323
	WN-182	PQ858675	PQ870169	PQ862324
	WN-181	PQ858676	PQ870170	PQ862325
	WN-180	PQ858677	PQ870171	PQ862326
	WN-140	PQ858680	PQ870172	PQ862327
	WN-139	PQ858681	PQ870173	PQ862328
	WN-138	PQ858682	PQ870174	PQ862329
	WN-137	PQ858683	PQ870175	PQ862330
	WN-136	PQ858684	PQ870176	PQ862331
	WN-107	PQ858685	PQ870177	PQ862332
	WN-104	PQ858686	PQ870178	PQ862333
	WN-95	PQ858657	PQ870179	PQ862334
	WN-94	PQ858658	PQ870180	PQ862335
	WN-86	PQ858659	PQ870181	PQ862336
	WN-85	PQ858660	PQ870182	PQ862337
	WN-80	PQ858661	PQ870183	PQ862338
	WN-78	PQ858662	PQ870184	PQ862339
	WN-77	PQ858663	PQ870185	PQ862340
	WN-70	PQ858664	PQ870186	PQ862341
	WN-51	PQ858671	PQ870189	PQ862342
	WN-56	PQ858668	PQ870188	PQ862343
	WN-61	PQ858665	PQ870187	PQ862344

**Table A6:** Phylogenetic tree partitions and evolutionary models selected of short segments of *Siphonaria* species.

Genes	Model
*12S rRNA*	TIM2+F+R3
*16S rRNA*	TIM2+F+R3
*COI* (p1)	HKY+F+R2
*COI* (p2)	TIM2+F+I+G4
*COI* (p3)	TVM+F+G4
*h3* (p1)	TN+F
*h3* (p2)	K2P+R2
*h3* (p3)	K2P+R2
